# TNPO1-Mediated Nuclear Import of FUBP1 Contributes to Tumor Immune Evasion by Increasing NRP1 Expression in Cervical Cancer

**DOI:** 10.1155/2021/9994004

**Published:** 2021-04-24

**Authors:** BiKang Yang, Jing Chen, YinCheng Teng

**Affiliations:** ^1^Department of Obstetrics and Gynecology, Shanghai Jiao Tong University Affiliated Sixth People's Hospital, Shanghai Jiao Tong University School of Medicine, Shanghai 200233, China; ^2^Department of Obstetrics and Gynecology, Shanghai Eighth People's Hospital Affiliated to Jiangsu University, Shanghai 200233, China

## Abstract

Far upstream element binding protein 1 (FUBP1), a DNA-binding protein, participates in diverse tumor-promoting behaviors by regulating the expression of oncogenes in the nucleus, but the underlying mechanisms remain to be elucidated. In the present study, we found that FUBP1 mRNA and protein expressions were markedly upregulated and closely linked with poor prognosis in cervical cancer. *In vitro*, functional experiments showed that knockdown of *FUBP1* inhibited CC cell proliferation and migration. Therefore, FUBP1 plays a prooncogenic function in CC progression. Further investigations for the first time demonstrated that nuclear localization of FUBP1 regulated the gene expression of immune checkpoint NRP1. Moreover, our work demonstrated that FUBP1 translocated into the nucleus which was mediated by interacting with Transportin-1 (TNPO1). Collectively, this study revealed that FUBP1 might be a potential therapeutic target for the restriction of tumor progression.

## 1. Introduction

FUBP1 (far upstream element binding protein 1) is an important regulator of transcription and translation that exerts its function by binding to the distal far upstream element (FUSE) [1]. The oncoprotein role of FUBP1 and overexpression of FUBP1 have been demonstrated in multiple types of cancers, such as hepatocellular carcinoma, neuroblastoma, myeloid leukemia, and endometrial cancer [2–5]. As a DNA helicase V, FUBP1 regulates the expression of downstream target genes, including *MYC*, by forming stable complexes with single-stranded DNA and promoting oncogenic processes, such as tumorigenesis and progression [6, 7]. Given that the nuclear localization of FUBP1 crucially affects the transcription of oncogenes, we speculated that blocking the nuclear import of FUBP1 suppresses cancer proliferation and becomes a potential target for cancer therapy.

During the immune response, immune system maintains self-tolerance or prevents side tissue damage using a series of immune checkpoints [8]. To further understand the complex tissue microenvironment (TME) under pathophysiological conditions, such as tumor TME, it is important to analyze immune checkpoint proteins and phenotypic markers. Classical immune checkpoint proteins PD-1 and CTLA-4 are upregulated in tumor-infiltrating T cells, and checkpoint blockade immunotherapy established a new approach in cancer treatment [9, 10]. As an unidentified immune checkpoint in T cells, blocking Neuropilin-1 (NRP1) can improve immunotherapy and prevent cancer recurrence [11]. NRP1 was originally identified as a neuronal and endothelial receptor that is required for normal embryonic development and angiogenesis [12]. NRP1 is also expressed in a variety of immune cell types involving in some immune functions [13]. NRP1 is upregulated in T_reg_ cells of cancer patients, suggesting that it may be a novel target of cancer immunotherapy.

Dysregulation of the nuclear-cytoplasmic transport of macromolecules is associated with many diseases, including cancer [14]. Nuclear-cytoplasmic translocation is responsible for regulating the physiological levels and temporal-spatial positions of tumor suppressors, oncoproteins, and other macromolecules, which are closely associated with tumorigenesis and drug resistance processes [15, 16]. Functional proteins with locating in the nucleus, such as transcription factors, are synthesized in the cytoplasm and enter the nucleus by interacting with importins to regulate gene expression and signal transduction [17, 18]. *TNPO1* encodes a nuclear import protein that participates in the nuclear transport of macromolecules, ciliary transport, and mitosis [19]. In addition, TNPO1 mediates the transcription factor Snail into the nucleus to inhibit the expression of E-cadherin and enhance the invasion of liver cancer cells [20]. Therefore, it is particularly urgent and important to explore the mechanisms of nuclear-cytoplasmic transport of macromolecules in cancers.

In the present study, the expression of *FUBP1* was markedly increased in CC tissue, and increased expression worsened the prognosis of CC patients. The biological experiments showed that FUBP1 promoted CC cell proliferation and migration. We further demonstrated FUNP1 regulated the gene transcription of immune checkpoint NRP1 and potentiates immune suppression. Moreover, we revealed for the first time a PY-NLS in the FUBP1 sequence and found that TNPO1 is responsible for the nuclear import of FUBP1.

## 2. Materials and Methods

### 2.1. Human CC Tissue Specimens

In this research, the clinical patient tissue microarray contained 40 paired cervical cancer and paracarcinoma tissue specimens from Shanghai Jiao Tong University Affiliated Sixth People's Hospital. All tissue specimens were confirmed by pathologist diagnosis. The informed consent was given to patients before this research.

### 2.2. Cell Culture

Human CC cells (MS751 and Siha) and HEK-293 cells were preserved in Shanghai Cancer Institute, Ren Ji Hospital, School of Medicine, Shanghai Jiao Tong University. These cells were all cultured in DMEM (GIBCO) and supplemented with 10% FBS and (*v*/*v*) penicillin/streptomycin at 37°C in an atmosphere containing 5%-CO2.

### 2.3. Immunohistochemical Staining

Clinical patient tissue microarray was bedded in paraffin for immunohistochemistry. IHC staining and score criteria were showed as previous research [21]. The primary antibody used was anti-FUBP1 (dilution 1 : 1000, ab181111, Abcam).

### 2.4. Quantitative Real-Time PCR

Total mRNA was extracted from cells using Trizol reagent (Takara) following the operating protocol. qRT-PCR was performed with SYBR Green Supermix (Bimake) on a 7500 RT-PCR system (Applied Biosystems). Reference gene 18S was utilized to normalization. Primer sets used for *FUBP1*, *NRP1*, *MYC*, and 18 s RNA examination were as follows: *FUBP1* forward 5′-GGAACAACACCTGATAGGATAGC-3′, *FUBP1* reverse 5′-GCCAGCCTGAACACTTCGTAG-3′; *NRP1* forward 5′-GGCGCTTTTCGCAACGATAAA -3′, *NRP1* reverse 5′-TCGCATTTTTCACTTGGGTGAT -3′; *MYC* forward 5′-ATGCCCCTCAACGTGAACTTC-3′, *MYC* reverse 5′-CGCAACATAGGATGGAGAGCA -3′; 18 s forward 5′-TGCGAGTACTCAACACCAACA-3′, 18 s reverse 5′-GCATATCTTCGGCCCACA-3′.

The formula RQ = 2 − ΔCT was utilized to calculate gene expression levels.

### 2.5. Small Interfering RNA

siRNAs against FUBP1 and TNPO1 were purchased from Gene Pharma (Shanghai, China). Transfection according to the manufacture's protocols uses Lipofectamine 3000. For FUBP1 siRNA: si*FUBP1*-1: 5-GGUGUUCGCAUUCAGUUUA-3, si*FUBP1*-2: 5-GGUGCUGACAAACCUCUUA-3. For TNPO1 siRNA: si*TNPO1*: 5-GUAGGACUCAAGCUCUAAU-3.

### 2.6. Western Blotting

Whole-cell lysates and separate nuclear/cytoplasmic fractions were extracted from cells according to routine protocols. Western blotting and coimmunoprecipitation were executed as preceding description [21]. The antibodies against GAPDH (60004-1-Ig), Flag-tag (20543-1-AP), *α*-tubulin (11224-1-AP), Lamin B1 (12987-1-AP), and GST-tag (HRP-66001) were purchased from ProteinTech. The antibodies against TNPO1 (ab10303, Abcam) and FUBP1 (ab181111, Abcam).

### 2.7. Cell Viability and Colony Formation Assay

MS751 and Siha cells were transfected with si-*FUBP1*-1, si-*FUBP1*-2, or siNC. CCK-8 assay and colony formation assay were executed as preceding description [21].

### 2.8. Edu Stain and Immunofluorescence Assay

MS751 and Siha cells, after transfected with si*FUBP1*-1, si*FUBP1*-2, or siNC 48 h and cotreatment with 100 *μ*L of EdU reagent for 4 h. Cells were fixed using 4% paraformaldehyde for 30 min and permeabilized with 0.3% Triton X-100. Immunofluorescence images were acquired by using the confocal microscope (Carl Zeiss, Germany) as the describe of protocol.

### 2.9. Plasmid DNA

The full-length sequence and mutant constructs of human FUBP1 were digestion into the 5′ NheI and 3′ NotI of pcDNA3.1-Flag-EGFP. PY-NLS constructs were generated into the 5′ BsrGI and 3′ EcoRI restriction sites of pcDNA3.1-GST-EGFP. The construct of M9M was digested into 5′ BsrGI and 3′ EcoRI of the pcDNA3.1 vector [22]. Plasmid DNAs were constructed and mutagenized using standard PCR-based methodologies, and all protein-coding regions that were generated were verified by DNA sequencing.

### 2.10. Statistical Analysis

Data were presented as the mean ± SD. The SPSS 19.0 and GraphPad Prism 8.0 software was employed for statistical analysis. Student's *t*-test was employed to analyze two groups of data. Values of *p* < 0.05 were considered statistically significant.

## 3. Results

### 3.1. FUBP1 Overexpression Is Correlated with Poor Prognosis in CC

**Figure 1:**
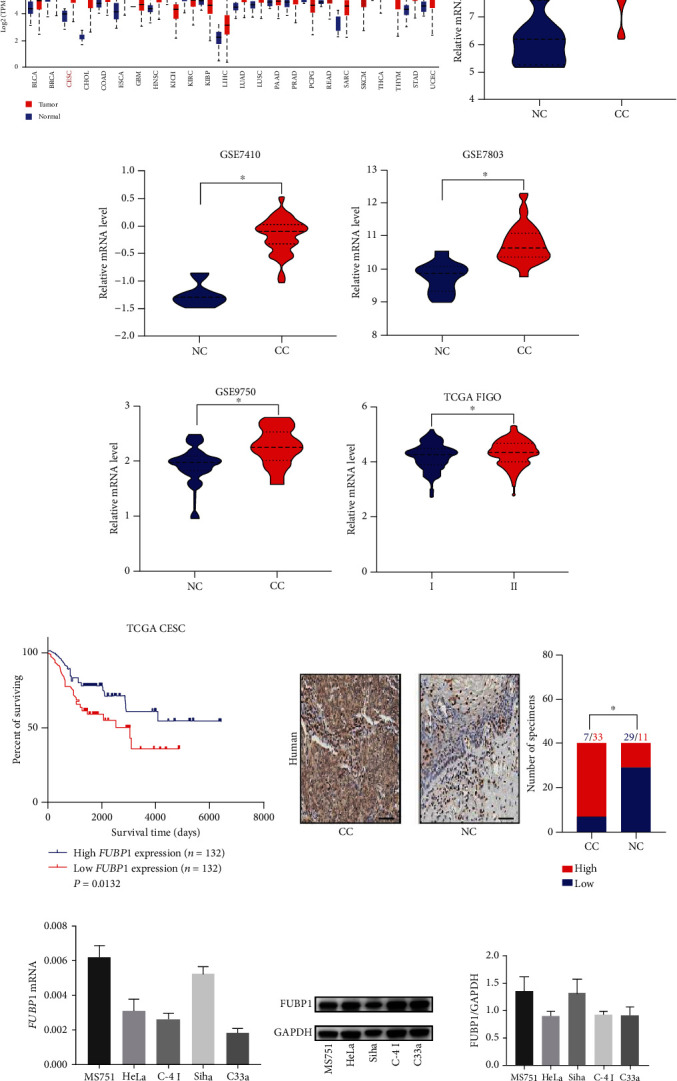
FUBP1 is upregulated and correlated with a poor prognosis in cervical cancer. (a) Analysis of *FUBP1* genetic expression across various human cancers using TCGA databases. (b–e) Expression profiles of FUBP1 in cervical cancer (CC) and normal cervix (NC) samples from GSE6791 (b), GSE7410 (c), GSE7803 (d), and GSE9750 (e) datasets. (f) Expression profiles of FUBP1 in FIGO from TCGA databases. (g) Kaplan-Meier analysis of the overall survival of patients with FUBP1 high or low expression level. (h) Representative immunohistochemical images and quantification analysis showing FUBP1 expression in CC and paracarcinoma samples from the sixth hospital. (i, j) The mRNA (i) and protein levels (j) in different CC cell lines. Scale bar: 50 *μ*m. Two-tailed *t*-test, ^∗^*p* < 0.05.

The genetic overexpression of *FUBP1* was demonstrated in a multitude of cancers by comparing pan-cancer gene expression, such as cervical cancer (CC), which indicates that *FUBP1* may act as an oncogene (Figure 1(a)). The expression profiles of *FUBP1* in the GSE6791 dataset showed that *FUBP1* expression was significantly upregulated in CC tissues compared with normal cervix tissues (Figure 1(b)). Moreover, we indicated that *FUBP1* expression was markedly increased in CC tissues by analyzing other GEO datasets (GSE9750, GSE7410, and GSE7803) (Figures 1(c)–1(e)). Meanwhile, high *FUBP1* mRNA expression was strongly correlated with tumor progression and poor survival in CC patients (Figures 1(f) and 1(g)). FUBP1 upregulation was further validated by immunohistochemical staining assay in a tissue microarray, which included 40 paired CC/paracarcinoma tissue specimens (Figure 1(h)). Overall, we speculated that *FUBP1* is a particular prooncogenic gene that selectively contributes to the progression of CC.

### 3.2. FUBP1 Promoted CC Cell Proliferation and Migration In Vitro

**Figure 2:**
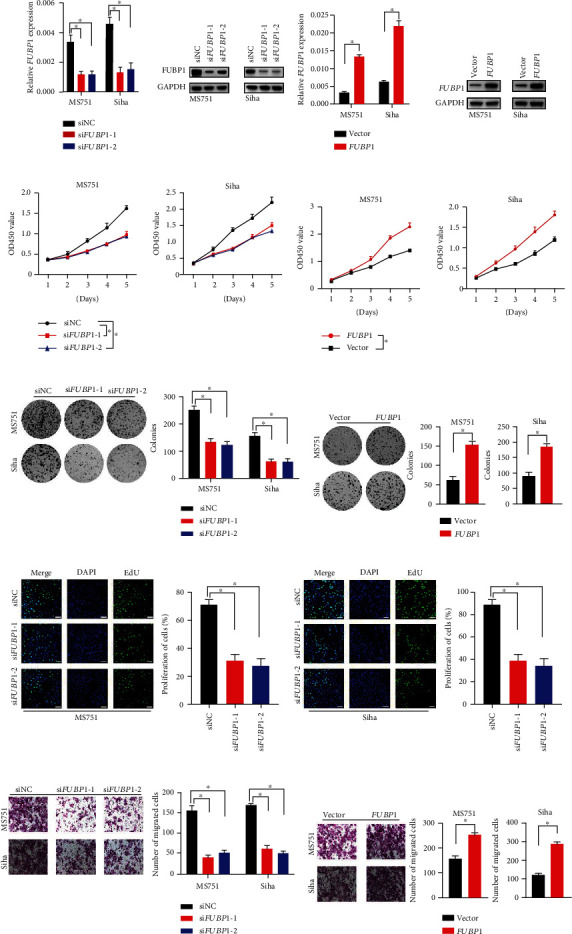
FUBP1 promotes the cell proliferation and migration of CC *in vitro.* (a, b) The mRNA and protein level in MS751 and Siha after FUBP1 knockdown or overexpression. (c, d) CCK-8 analysis of MS751 and Siha cell viability after FUBP1 knockdown or overexpression. (e, f) Representative colony formation and quantification number of colonies after FUBP1 knockdown or overexpression. (g) Representative EdU staining image and quantification ratio of proliferation cells after FUBP1 knockdown. (h, i) Representative transwell assay and quantification analysis after FUBP1 knockdown or overexpression. Error bars represent mean ± standard error of mean. Scar bar: 50 *μ*m. Two-tailed *t*-test, ^∗^*p* < 0.05.

To validate the roles of FUBP1 in CC development, two CC cell lines with higher FUBP1 mRNA and protein levels, MS751 and Siha, were picked up (Figures 1(i) and 1(j)). Knockdown of FUBP1 and overexpression of FUBP1 were confirmed by quantitative real-time PCR and immunoblot analysis (Figures 2(a) and 2(b)). To analyze the effect of FUBP1 on CC cell proliferation, a CCK-8 assay was performed. Knockdown of *FUBP1* reduced cell viability (Figure 2(c)). In contrast, the overexpression of FUBP1 markedly enhanced cell viability (Figure 2(d)). In line with these findings, the clonogenic assay was utilized to validate the above results (Figures 2(e) and 2(f)). We also performed an EdU staining assay to detect cell proliferation. Consistently, the percentage of EdU-positive cells decreased after FUBP1 knockdown (Figure 2(g)). Meanwhile, we found that knockdown of FUBP1 reduced the migratory capability of MS751 and Siha cells, while an enhanced migratory capability was demonstrated in the FUBP1 overexpression groups (Figures 2(h) and 2(i)). Collectively, these data indicated that FUBP1 promotes CC cell proliferation and migration.

### 3.3. FUBP1 Promoted the NRP1 Expression and Contributed to Tumor Immune Evasion

**Figure 3:**
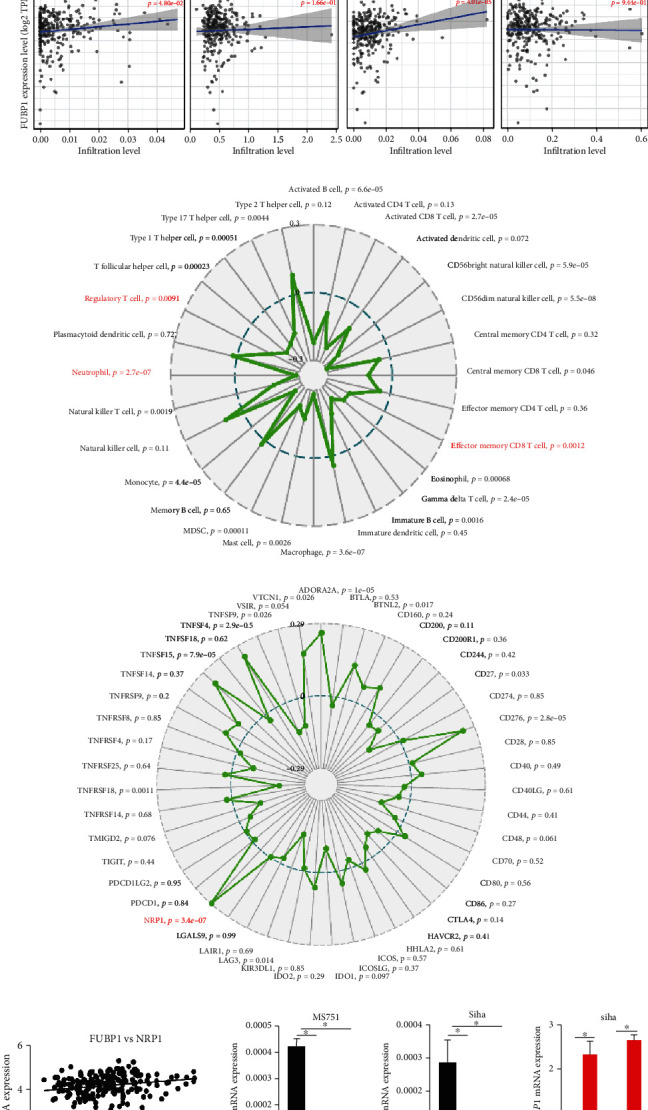
FUBP1 contributed to tumor immune evasion by regulating NRP1 expression. (a) The abundances of immune infiltrates are estimated by TIMER algorithm. (b, c) Gene-immune analysis of FUBP1 in CC conducted on Sanger box. (d) The correlation between expression levels of FUBP1 and NRP1 in CC. (e) The mRNA level of NRP1 in MS751 and Siha after FUBP1 knockdown or overexpression. Error bars represent mean ± standard error of mean. Two-tailed *t*-test, ^∗^*p* < 0.05.

Immunotherapy has revealed promise in solid tumor treatment. To further investigate the function of FUBP1 in CC progression, we first utilized expression of FUBP1 to analysis the infiltration level of different immune cells in CC tissues by TIMER database. As expected, the expression of FUBP1 significantly positive association with Neutrophil, T_reg_, and CD8+ cells infiltration (Figure 3(a)). Moreover, we performed the Gene-Immune Analysis using Sanger box (http://sangerbox.com/Index). These analysis results demonstrated that the overexpression of FUBP1 is correlated with Memory CD8+ T cell, Regulatory T cell, and Neutrophil immune pathways (Figure 3(b)). We also found that the high expression of FUBP1 is closely associated with a various of expression of immune checkpoints, especially NRP1 (Figure 3(c)). NRP1 as an immune checkpoint plays a crucial role in limiting long-term antitumor immunity. We measured the mRNA levels of FUBP1 and NRP1 in the CESC TCGA database and analyzed their correlations. A positive correlation between FUBP1 and NRP1 was detected in CC patients (Figure 3(d)). Furthermore, knockdown of FUBP1 remarkedly suppressed NRP1 expression compared to control groups in MS751 and Siha cells (Figure 3(e)). In contrast, the overexpression of FUBP1 markedly enhanced the expression of NRP1.

### 3.4. The PY-NLS Motif of FUBP1 Is Necessary and Sufficient for Nuclear Import

**Figure 4:**
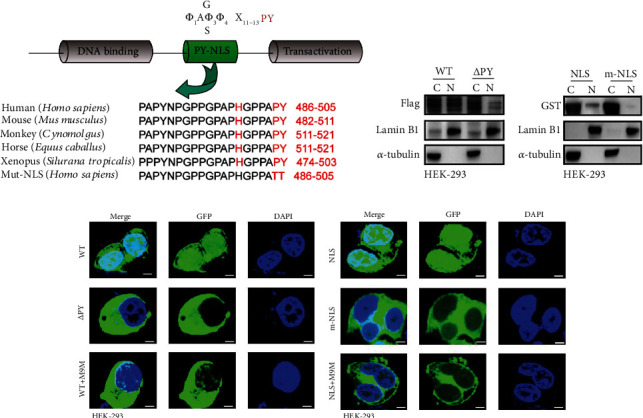
The PY-NLS motif is responsible for the nuclear import of FUBP1. (a) The cylinder shows the domain of the PY-NLS motif and function of FUBP1. Alignment revealing the FUBP1 PY-NLS motif from various mammalian species. (b) Immunoblots shows the FUBP1 and mutant levels in the nucleoplasm and cytoplasm of HEK-293 cell. (c) Immunoblots shows the PY-NLS and mutant levels in the nucleoplasm and cytoplasm. (d) Confocal microscopy of FUBP1 and mutant levels. (e) Confocal microscopy of PY-NLS and mutant levels. Scar bar 20 *μ*m.

To regulate the expression of NRP1, the nuclear import of FUBP1 requires nuclear localization signal (NLS) modulation. We noted the nonclassical PY-NLS motif, which was recognized by Transportin 1 (TNPO1), within amino acids FUBP1 486-505, and the motif is highly conserved in various mammalian species (Figure 4(a)). To further investigate whether the PY-NLS within the C-terminal domain of FUBP1 is required for nuclear import, we generated a deletion mutant lacking the PY-NLS motif (486-505). Immunoblot analysis of separate nuclear/cytoplasmic fractions and immunofluorescence assay showed nuclear accumulation of WT FUBP1 and redistribution of the deletion mutant to the cytoplasm (Figures 4(b) and 4(d)). To test whether the PY-NLS motif of FUBP1 is not only necessary but also sufficient for active nuclear import, the PY-NLS domain and its point mutant (P504T/Y505T) were inserted into the C-terminus of the cytosolic reporter protein GST-GFP (∼55 kDa). In contrast to PY-NLS, results showed that the point mutant was mostly located in the cytoplasm (Figures 4(c) and 4(e)). Moreover, the nuclear localization of WT and PY-NLS were dramatically decreased when the TNPO1-specific inhibitor small peptide (M9M) was transfected (Figures 4(d) and 4(e)) [23]. Taken together, these results demonstrate that the PY-NLS within the C-terminus of FUBP1 is necessary and sufficient for active nuclear import.

### 3.5. Karyopherin TNPO1 Modulates the Nuclear Import of FUBP1

**Figure 5:**
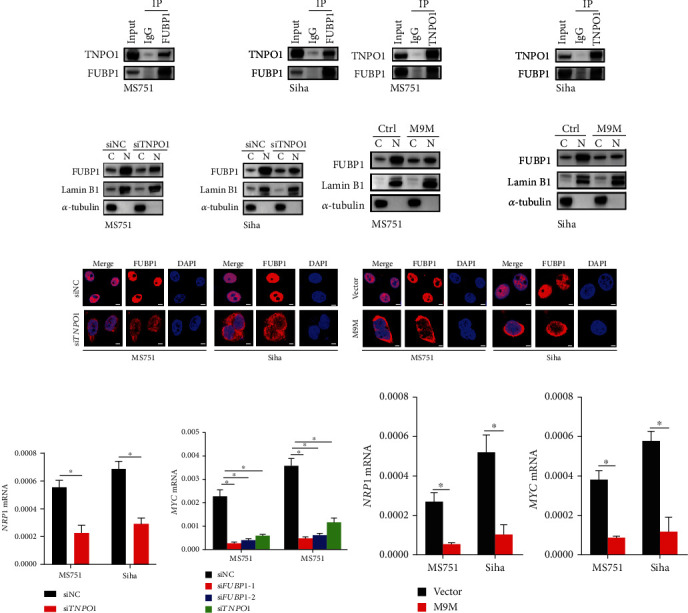
Karyopherin TNPO1 mediates nuclear import of FUBP1. (a) Immunoblots of the TNPO1 levels following immunoprecipitation of FUBP1 in MS751 and Siha cells. (b) Immunoblots of the FUBP1 levels following immunoprecipitation of TNPO1. (c, d) Immunoblots shows the FUBP1 levels in the nucleoplasm and cytoplasm after transfecting with siTNPO1 or M9M. (e, f) Confocal microscopy shows the FUBP1 protein after transfecting with siTNPO1 or M9M. (g, h) The mRNA level of *NRP1* and *MYC* after FUBP1 or TNPO1 knockdown. (i) The mRNA level of *NRP1* and *MYC* after M9M expression. Error bars represent mean ± standard error of mean. Scar bar: 20 *μ*m. Two-tailed *t*-test, ^∗^*p* < 0.05.

The sequence of FUBP1 contained a PY-NLS (proline-tyrosine) motif, which was recognized by Transportin 1 (TNPO1). Therefore, our results demonstrated that FUBP1 was readily coimmunoprecipitated with TNPO1 in MS751 and Siha cells and vice versa (Figures 5(a) and 5(b)). Moreover, immunoblot analysis of separate nuclear/cytoplasmic fractions and immunofluorescence assay indicated that the nuclear localization of FUBP1 was decreased when TNPO1 was knocked down (Figures 5(c) and 5(e)). Meanwhile, the nuclear localization of FUBP1 was decreased when the M9M construct was expressed in cancer cells (Figures 5(d) and 5(f)). To further explore the effects of blocking nuclear import on the transcription-correlation function of FUBP1, we examined the expression of *NRP1* and the downstream genes, such as *MYC*. As expected, knockdown of TNPO1 or M9M also remarkedly suppressed the expression of *NRP1* and *MYC* (Figure 5(i)). The analysis results also revealed that the effects detected by si*FUBP1s* were similar (Figures 5(g) and 5(h)), as shown in previous studies [7]. Collectively, our work demonstrated that TNPO1 mediates the nuclear import of FUBP1 and regulates transcription-correlation function.

## 4. Discussion

Human far upstream element binding protein 1 (FUBP1) is an important regulator of gene transcription and translation [7]. As a transcription-associated DNA helicase, dysregulation of the *FUBP1* gene is a frequently occurring event in a multitude of malignancies and is associated with tumorigenesis and progression, and FUBP1 has increasingly become a novel pharmacological target for cancer treatment [24]. This work intends to investigate the biological functions and molecular mechanisms of *FUBP1* in CC progression.

A series of transcription-associated regulators have demonstrated that these genes exhibit both tumorigenic and antitumorigenic functions in different cancers [25]. The “double-agent” functions of the *FUBP1* gene have been identified in a variety of cancers; for example, genomic loss-of-function mutations are linked with poor survival in oligodendrogliomas, suggesting a tumor-suppressive function of FUBP1 [26]. In contrast, in other tumors, including hepatocellular carcinoma and ovarian cancer, the more general genomic alteration of *FUBP1* is excessive expression, which is often inversely correlated with overall survival [2, 27]. In the present study, widespread computational bioinformatic analysis from some independent databases and TAMs results demonstrated that the expression of FUBP1 was significantly increased in CC and was associated with poor prognosis. Moreover, our work demonstrated that knockdown of *FUBP1* suppressed the proliferation and migration of CC cells. Therefore, we suggest that *FUBP1* may play an oncogenic function in CC progression.

The dysregulated expression of ligands and oncogenes contributes to tumor immune evasion by activating immune checkpoints during cancer progression and metastasis [28]. NRP1 exerts coreceptor function for LAP-TGF-*β* by binding the Glycoprotein A repetitions predominant, which links to poor tumor immunity, in breast cancer [29]. Previous studies demonstrate that an intratumoral expression of NRP-1/Sema3A blocking biologicals increases antitumor immunity [30]. Checkpoint blockade immunotherapy (ICB) has revolutionized tumor-treatment, but just a small percentage (10%-30%) of cancer patients establish lasting clinical responses [31]. In the harsh tumor microenvironments, the immunoregulatory receptor Neuropilin-1 (NRP1) is very important to maintain the function, integrity, and survival of intertumoral regulatory T cells (T_reg_ cells) [32]. Unlike other immune checkpoints, such as PD1 and CTLA4, NRP1 can not only enhances the function of T_reg_ cells but also inhibits and limits CD8+ T cell memory response during an antitumor immune response [33]. In this study, we found that the expression of FUBP1 associated with many immune checkpoint proteins, and NRP1 is the most obvious one. Knockdown of FUBP1 significantly reduced the expression of NRP1 in CC cells. Whether FUBP1 is associated with NRP1 expression in tumor-infiltrated lymphocytes remains more discussion. These results indicated that FUBP1 may be contributed to the regulation of tumor immune inhibitory by increasing the expression of NRP1 in CC cells.

FUBP1 interacts with single-stranded DNA (ssDNA) and forms a stable complex through its four K-homology (KH) motifs [34]. To ensure accurate DNA-binding transcription-correlation function of FUBP1, FUBP1 is commonly enriched in the nucleus. FUBP1, as a macromolecular substance, with a mass of 67 kD, and the nuclear-cytoplasmic transport of the FUBP1 protein requires binding to karyopherin-*β* proteins [35]. In the present study, we found a nonclassical PY-NLS motif within amino acids FUBP1 486-505. Our mutational analysis suggests that the PY-NLS motif of FUBP1 is necessary and sufficiently required for active nuclear import. Moreover, the immunoblot analysis and immunofluorescence results revealed for the first time a new molecular mechanism for the nuclear-cytoplasmic transport of FUBP1, which was modulated by TNPO1. FUBP1 also endorses oncogenic functions by activating the transcription of its target oncogenes. Overexpression of *FUBP1* alters the expression of the oncogene *MYC* to promote cancer cell proliferation by interacting with FUBP interacting repressor (FIR) and transcription factor IIH (TFIIH) [36, 37]. In the present study, we found that knockdown of *FUBP1* suppressed the expression of *MYC*. Moreover, we further confirmed the expression change of *MYC* and *NRP1* associated with knockdown of *TNPO1* to block the nuclear import of FUBP1. Based on the above results, we demonstrated for the first time that TNPO1 mediated the nuclear import of FUBP1 and then confirmed that FUBP1 regulated gene transcription.

## 5. Conclusion

The present study demonstrated that FUBP1 was overexpressed and associated with poor prognosis in CC. Knockdown of FUBP1 impaired CC cell proliferation and migration. Further studies demonstrated that the nuclear localization of FUBP1 contributed to tumor immune evasion by regulating the expression of NRP1. Moreover, we found the nuclear import of FUBP1 was mediated by TNPO1 and contributed to regulating the gene transcription of oncogenes. These findings strongly suggest that FUBP1 maybe become a novel therapeutic strategy in CC treatment.

## Data Availability

The data used to support the of this study were supplied by Yincheng Teng under license and so cannot be made freely available. Requests for access to these data should be considered by the corresponding author.

## References

[1] Zhang J., Chen Q. M. (2013). Far upstream element binding protein 1: a commander of transcription, translation and beyond. *Oncogene*.

[2] Samarin J., Laketa V., Malz M. (2016). PI3K/AKT/mTOR-dependent stabilization of oncogenic far-upstream element binding proteins in hepatocellular carcinoma cells. *Hepatology*.

[3] Jiang P., Huang M., Qi W. (2019). FUBP1 promotes neuroblastoma proliferation via enhancing glycolysis-a new possible marker of malignancy for neuroblastoma. *Journal of Experimental & Clinical Cancer Research*.

[4] Hoang V. T., Verma D., Godavarthy P. S. (2019). The transcriptional regulator FUBP1 influences disease outcome in murine and human myeloid leukemia. *Leukemia*.

[5] Han T., Wu Y., Hu X. (2020). NORAD orchestrates endometrial cancer progression by sequestering FUBP1 nuclear localization to promote cell apoptosis. *Cell Death & Disease*.

[6] Zhong Q., Liu Z. H., Lin Z. R. (2018). The RARS-MAD1L1 fusion gene induces cancer stem cell-like properties and therapeutic resistance in nasopharyngeal carcinoma. *Clinical Cancer Research*.

[7] Debaize L., Troadec M. B. (2019). The master regulator FUBP1: its emerging role in normal cell function and malignant development. *Cellular and Molecular Life Sciences*.

[8] Chuckran C. A., Liu C., Bruno T. C., Workman C. J., Vignali D. A. (2020). Neuropilin-1: a checkpoint target with unique implications for cancer immunology and immunotherapy. *Journal for ImmunoTherapy of Cancer*.

[9] O’Day S. J., Maio M., Chiarion-Sileni V. (2010). Efficacy and safety of ipilimumab monotherapy in patients with pretreated advanced melanoma: a multicenter single-arm phase II study. *Annals of Oncology*.

[10] Brahmer J. R., Tykodi S. S., Chow L. Q. (2012). Safety and activity of anti-PD-L1 antibody in patients with advanced cancer. *The New England Journal of Medicine*.

[11] Liu C., Somasundaram A., Manne S. (2020). Neuropilin-1 is a T cell memory checkpoint limiting long-term antitumor immunity. *Nature Immunology*.

[12] Kawasaki T., Kitsukawa T., Bekku Y. (1999). A requirement for neuropilin-1 in embryonic vessel formation. *Development*.

[13] Acharya N., Anderson A. C. (2020). NRP1 cripples immunological memory. *Nature Immunology*.

[14] Cagatay T., Chook Y. M. (2018). Karyopherins in cancer. *Current Opinion in Cell Biology*.

[15] Hanahan D., Weinberg R. A. (2011). Hallmarks of cancer: the next generation. *Cell*.

[16] Nachmias B., Schimmer A. D. (2020). Targeting nuclear import and export in hematological malignancies. *Leukemia*.

[17] Hoelz A., Debler E. W., Blobel G. (2011). The structure of the nuclear pore complex. *Annual Review of Biochemistry*.

[18] Marvaldi L., Panayotis N., Alber S. (2020). Importin *α*3 regulates chronic pain pathways in peripheral sensory neurons. *Science*.

[19] Twyffels L., Gueydan C., Kruys V. (2014). Transportin-1 and Transportin-2: protein nuclear import and beyond. *FEBS Letters*.

[20] Zhu M., Yin F., Fan X. (2015). Decreased TIP30 promotes Snail-mediated epithelial-mesenchymal transition and tumor-initiating properties in hepatocellular carcinoma. *Oncogene*.

[21] Yang B., Chen J., Teng Y. (2021). CDK12 promotes cervical cancer progression through enhancing macrophage infiltration. *Journal of Immunology Research*.

[22] Cansizoglu A. E., Lee B. J., Zhang Z. C., Fontoura B. M., Chook Y. M. (2007). Structure-based design of a pathway-specific nuclear import inhibitor. *Nature Structural & Molecular Biology*.

[23] Iijima M., Suzuki M., Tanabe A., Nishimura A., Yamada M. (2006). Two motifs essential for nuclear import of the hnRNP A1 nucleocytoplasmic shuttling sequence M9 core. *FEBS Letters*.

[24] Debaize L., Jakobczyk H., Avner S. (2018). Interplay between transcription regulators RUNX1 and FUBP1 activates an enhancer of the oncogene c-KIT and amplifies cell proliferation. *Nucleic Acids Research*.

[25] Shen L., Shi Q., Wang W. (2018). Double agents: genes with both oncogenic and tumor-suppressor functions. *Oncogene*.

[26] Thomas A. A., Abrey L. E., Terziev R. (2017). Multicenter phase II study of temozolomide and myeloablative chemotherapy with autologous stem cell transplant for newly diagnosed anaplastic oligodendroglioma. *Neuro-Oncology*.

[27] Ma Y., Wang X., Qiu C. (2021). Using protein microarray to identify and evaluate autoantibodies to tumor-associated antigens in ovarian cancer. *Cancer Science*.

[28] Casazza A., Laoui D., Wenes M. (2013). Impeding macrophage entry into hypoxic tumor areas by Sema3A/Nrp1 signaling blockade inhibits angiogenesis and restores antitumor immunity. *Cancer Cell*.

[29] Glinka Y., Stoilova S., Mohammed N., Prud'homme G. J. (2011). Neuropilin-1 exerts co-receptor function for TGF-beta-1 on the membrane of cancer cells and enhances responses to both latent and active TGF-beta. *Carcinogenesis*.

[30] de Vlaeminck Y., Bonelli S., Awad R. M. (2020). Targeting neuropilin-1 with nanobodies reduces colorectal carcinoma development. *Cancers*.

[31] Ferris R. L., Blumenschein G., Fayette J. (2016). Nivolumab for recurrent squamous-cell carcinoma of the head and neck. *The New England Journal of Medicine*.

[32] Overacre-Delgoffe A. E., Chikina M., Dadey R. E. (2017). Interferon-*γ* drives T reg fragility to promote anti-tumor immunity. *Cell*.

[33] Leclerc M., Voilin E., Gros G. (2019). Regulation of antitumour CD8 T-cell immunity and checkpoint blockade immunotherapy by Neuropilin-1. *Nature Communications*.

[34] Valverde R., Edwards L., Regan L. (2008). Structure and function of KH domains. *The FEBS Journal*.

[35] Chien H. L., Liao C. L., Lin Y. L. (2011). FUSE binding protein 1 interacts with untranslated regions of Japanese encephalitis virus RNA and negatively regulates viral replication. *Journal of Virology*.

[36] Braddock D. T., Louis J. M., Baber J. L., Levens D., Clore G. M. (2002). Structure and dynamics of KH domains from FBP bound to single-stranded DNA. *Nature*.

[37] Crichlow G. V., Zhou H., Hsiao H. H. (2008). Dimerization of FIR upon FUSE DNA binding suggests a mechanism of c-myc inhibition. *The EMBO Journal*.

